# The Effect of Changes in Lower Incisor Inclination on Gingival Recession

**DOI:** 10.1155/2015/193206

**Published:** 2015-04-16

**Authors:** Gulen Kamak, Hasan Kamak, Hakan Keklik, Hakan Gurcan Gurel

**Affiliations:** ^1^Department of Periodontology, Faculty of Dentistry, Kırıkkale University, 71100 Kırıkkale, Turkey; ^2^Department of Orthodontics, Faculty of Dentistry, Kırıkkale University, 71100 Kırıkkale, Turkey; ^3^Private Practice, 1386 Street, No. 7, Alsancak, 35220 İzmir, Turkey

## Abstract

*Aim*. Orthodontic treatment may promote development of recessions. The mechanism by which orthodontic treatment influences occurrence of recessions remains unclear. The aim of this study was to test the hypothesis that a change of mandibular incisor inclination promotes development of labial gingival recessions. *Materials and Methods*. The study sample comprised dental casts and lateral cephalograms obtained from 109 subjects before orthodontic treatment (Tb) and after orthodontic treatment (Ta). Depending on the change of lower incisor inclination during treatment, the subjects were divided into three groups: Retroclination (R), Stable Position (S), and Proclination (P). The presence of gingival recessions of mandibular incisors and clinical crown heights were assessed on plaster models. *Results and Conclusions*. From Tb to Ta, Inc_Incl showed a statistically significant change in the R, P, and S groups (*p* < 0.05). Increase of clinical crown heights of the lower incisors (42, 4, and 31) was not statistically significant in any group. The only statistically significant intergroup difference was the greater increase of the clinical crown height of tooth number 32 in the P group in comparison with the R group (*p* = 0.049). The change of lower incisor inclination during treatment did not lead to development of labial gingival recessions in the study sample.

## 1. Introduction

Gingival recession is defined as the apical shift of the free gingival margin throughout the labial, lingual, or interproximal root surface [1]. From the clinical standpoint gingival recession is measured as the distance from CEJ to the most apical extension of gingival margin. Severity and prevalence of gingival recession increase and its presence was reported in more than 90 per cent of adults aged 50 years and above [2]. The labial aspect of the mandibular incisors and maxillary molars is being most frequently affected [3]. This clinical condition may result in dentin hypersensitivity, root caries, and esthetically unfavourable effects [4].

There are many causal effects in development of gingival recession, that is, tooth brushing trauma, destructive periodontal disease, tooth malpositioning, and destructive periodontal disease; tooth malpositioning, alveolar bone dehiscence, thin and delicate marginal tissue covering a nonvascularized root surface, high muscle attachment and frenal pull, and occlusal trauma; lip piercing and iatrogenic factors related to reconstructive, conservative periodontologic, orthodontic, or prosthetics treatment [5].

Controversy exists in the literature between the role of orthodontic treatment and gingival recession. Whilst movement of teeth outside the alveolar bone has been reported as a risk factor for gingival recession, others have not found such association [6–8]. Thus, the aim of this study was to test the hypothesis that a change of mandibular incisor inclination promotes development of labial gingival recessions.

## 2. Materials and Methods

The study sample comprised dental casts and lateral cephalograms obtained from orthodontic patients before (Tb) and after (Ta) the orthodontic treatment (Table 1). The number of randomly selected models was 109. Intraoral photographs of patients were examined and subjects with any missing tooth, with gingival recession, and with any congenital deformities such as cleft palate were excluded from the study and all of the patients had good oral hygiene and healthy gingival tissues. Only radiographic and plaster dental casts of good quality were included in our study. Information on gender, age, and systemic health at Tb and Ta was obtained from the patient files. Approval from the ethics committee was not required fort his retrospective study.

All subjects were treated with upper and lower straight-wire appliances for at least 12 months (mean treatment time: 1.80 ± 0.64). All pretreatment dental casts and lateral cephalographs were taken within one month prior to the start of the orthodontic treatment. The posttreatment records were taken on the day the active orthodontic appliances were removed. All radiographs were taken by an experienced X-ray technician using an orthopantomograph (Planmeca Proline CC 2002, Helsinki, Finland) with a magnification factor of 1.2.

All measurements on cephalograms and models were made by the same investigator (HKe) with an electronic caliper (MarCal 16 ER, Mahr GmbH, Göttingen, Germany) with an accuracy of 0.01 mm. Four weeks after the first set of measurements, 40 study records were randomly selected and measured again, and intraclass coefficients were calculated to estimate the method error. The coefficient for all measurements was between 0.93 and 0.98 and was considered acceptable.

**Figure 1:**
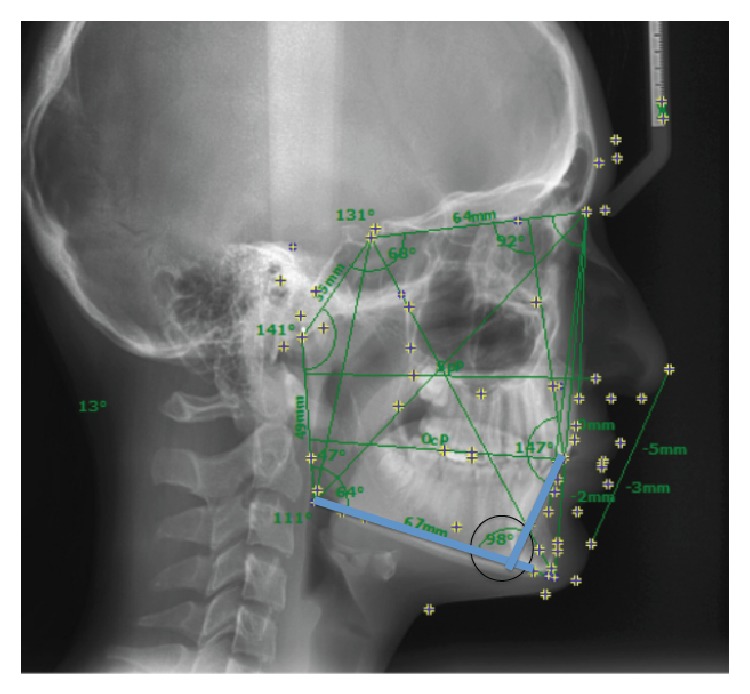
The degree of lower incisor inclination was measured by calculating the angle between mandibular plane (MP) and the long axis of most forward lower incisor on lateral cephalometric radiographs.

**Figure 2:**
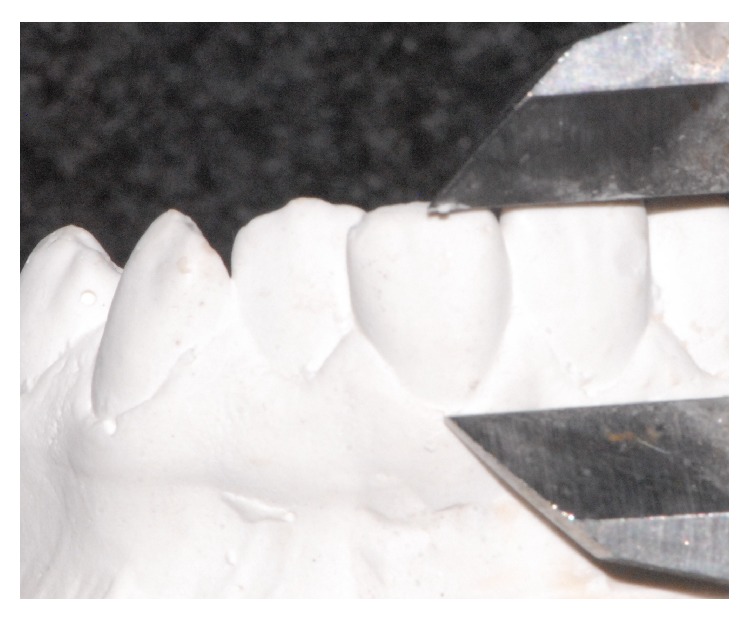
Before the orthodontic treatment, crown height was assessed with a digital caliper by measuring the distance between the most coronal point of incisor margin and the most apical point of gingival margin.

**Figure 3:**
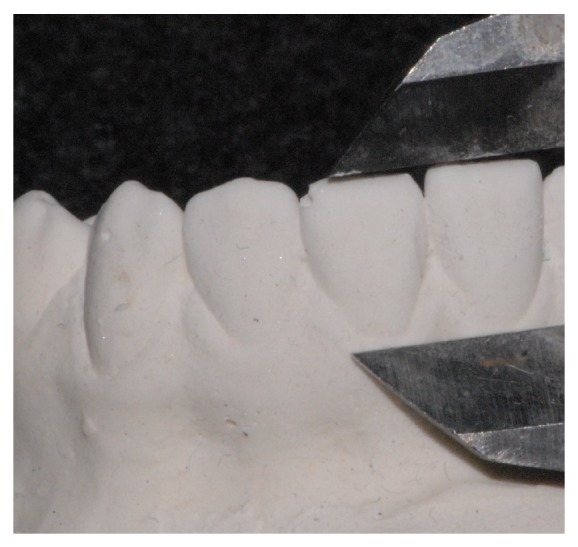
After the orthodontic treatment, crown height was assessed with a digital caliper by measuring the distance between the most coronal point of incisor margin and the most apical point of gingival margin.

The angle between mandibular plane (MP) and the long axis of most forward lower incisor (lower incisor inclination) was measured on Tb and Ta lateral cephalometric radiographs (Figure 1). Depending on the change of lower incisor inclination during treatment [Inc (Tb to Ta)], the subjects were divided into three groups: Retroclination (R) (*N* = 32; Inc (Tb to Ta) ≤ –1°), Stable Position (S) (*N* = 13; Inc (Tb to Ta) > –1° and ≤ 1°), and Proclination (P) (*N* = 64; Inc (Tb to Ta) > 1°). The distances between the incisal edges and the deepest points of the curvature of the vestibulogingival margin of all four mandibular incisors, corresponding to the “clinical crown heights,” were measured on the plaster models made at Tb and Ta (Figures 2 and 3). The amounts of labial gingival recessions were measured by extracting Tb clinical crown height from Ta crown height (clinical crown heights at Ta − clinical crown heights at Tb).

### 2.1. Statistical Methods

All descriptive and comparative statistical analyses were performed using the SPSS software package (Statistical Package for Social Sciences, version 15.0, SPSS Inc., Chicago, IL, USA). The Kolmogorov-Smirnov test was performed to test for the normality of age, treatment time, and inclination parameters. Then, these parameters were analyzed using the one-way ANOVA and Tukey post hoc tests for the parameters showing normal distribution [treatment time (Tb to Ta), Inc at Tb, Inc at Ta, and Inc (Tb to Ta)] and the Kruskal-Wallis test for the parameters showing nonnormal distribution (age at Tb and age at Ta). The significance level was set at *p* < 0.05 for all tests.

## 3. Results

Inc at Tb was greatest in the S group (97.23°) and smallest in the P group (90.42°) whereas Inc at Ta was greatest in the S group (97.69°) and smallest in the R group (92.63°). Inc (Tb to Ta) changed statistically significantly in the R, P, and S groups (*p* < 0.001) (Table 2).

Increase in clinical crown heights of the lower incisors (42,41,31) was not statistically significant in groups. The only statistically significant intergroup difference was the larger increase of the clinical crown height of tooth number 32 in the P group in comparison with the R group (*p* = 0.049) (Table 3). Of all the teeth we evaluated, significant (*p* = 0.049) recession was detected only in tooth number 32 in 2 patients and both recession cases were Miller Class I. Therefore, the most prevalent gingival recession was found to be Miller Class I. During the examination of incisal papilla on the casts, no change was observed.

## 4. Discussion

A number of predisposing and precipitating factors are considered important identifiable risk factors for gingival recession in relation to orthodontic treatment. Predisposing factors include anatomical and morphological characteristics, such as alveolar bone dehiscence, gingival biotype, skeletal pattern, narrow symphysis, and ectopic tooth eruption or morphology. Precipitating factors lead to an acceleration of the defect, such as traumatic tooth brushing, traumatic overbite, age, smoking, parafunctional habits, pregnancy, and piercing. In addition and perhaps equally important are inappropriate treatment mechanics, such as arch expansion, with excessive proclination, and the use of RME in adult patients. Care should also be taken when decompensating a Class III incisor relationship in preparation for surgery and aligning ectopic/transposed teeth [9].

Gingival recessions may compromise therapeutical outcome because they may adversely affect dentofacial aesthetics or cause tooth hypersensitivity. Although their aetiology is not clear, occurrence of gingival recessions may be associated with past orthodontic treatment [10]. Thus, the aim of the present study was to identify a possible relationship between the change of inclination of lower incisors during treatment and development of gingival recessions in the area of mandibular incisors.

The results of the present study indicate that neither changing the inclination of mandibular incisors nor maintaining them in the original positions during orthodontic treatment has any influence on the development of gingival recessions in the mandibular incisor region. Although we found that the increase of the clinical crown height in tooth number 32 in the SP group was larger than in the RC group, the difference was limited to only one tooth and the change of clinical crown heights of the remaining incisors was comparable. Therefore, the present findings concur with the results of previous studies investigating the possible correlation with mandibular incisor inclination and gingival recession [7, 8, 11, 12].

Several other studies, however, found the association between a change of inclination of lower incisors and increased risk of gingival recessions [9, 13–15]. The disagreement between our findings and the results of these studies can be explained by inclusion of subjects with recessions to the study group and evaluation of patients with Class III malocclusion who might have had thinner gingiva, more prone to recessions. Yared et al. observed that free gingival margins with a thickness <0.5 mm presented greater and more severe recession associated with mandibular central incisors [16]. Kao and Pasquinelli classified the periodontal biotype as thin or thick. Thin biotypes present a thin underlying bone, characterized by bony dehiscence and fenestration, which reacts to insults and disease with gingival recession [17]. The presence of a thin biotype was identified as a possible predictor of gingival recession in a study by Melsen and Allais [9].

Clinical attachment level refers to the distance from enamel cement line to the bottom of the clinical periodontal pocket and is an important parameter in investigating the recessions. As a result of doing the present study on clusters, clinical attachment levels could not be examined. Also this can be considered as a limitation of the present study.

## 5. Conclusion

Based on the findings of this study, it can be concluded that the change of inclination of lower incisors during orthodontic treatment did not lead to development of labial recessions in this study sample (Table 4). Thus, the hypothesis was rejected.

## Figures and Tables

**Table 1:** Distribution of data according to the gender and inclination groups.

	*N*	%
Gender		
Female	70	64.2
Male	39	35.8
Inclination groups		
Retroclination	32	29.4
Stable Position	13	11.9
Proclination	64	58.7
Total	**109**	**100**

**Table 2:** Characteristics of the inclination groups.

	Retroclination		Stable Position		Proclination					
	(R) (*N*: 32)		(S) (*N*: 13)		(P) (*N*: 64)		*p* value	Paired differences		
	Mean	Std. deviation	Mean	Std. deviation	Mean	Std. deviation				
Age at Tb	14.20	3.67	12.59	1.49	14.58	4.30	0.192	NS		
Age at Ta	15.96	3.71	14.35	1.42	16.39	4.23	0.211	NS		
Treatment time (Tb to Ta)	1.76	0.53	1.77	0.91	1.81	0.64	0.911	NS		

								*p* value		
								R − S	R − P	S − P

Inc at Tb	96.72	6.51	97.23	4.89	90.42	5.48	0.000	NS	0.000^∗∗^	0.000^∗∗^
Inc at Ta	92.63	6.20	97.69	4.96	96.23	5.72	0.006	0.024^∗^	0.013^∗^	NS
Inc (Tb to Ta)	−4.09	2.75	0.46	0.52	5.81	3.35	0.000	0.000^∗∗^	0.000^∗∗^	0.000^∗∗^

NS: not significant, ^∗^ *p* < 0.05, and ^∗∗^ *p* < 0.001.

One-way ANOVA, post hoc Tukey and Kruskal-Wallis test.

**Table 3:** The mean increase (mm) of clinical crown height of lower incisors (gingival recession) after treatment (from TB to TA).

Tooth number	Retroclination (R)		Stable Position (S)		Proclination (P)		*p* value
	Mean	Std. deviation	Mean	Std. deviation	Mean	Std. deviation	
42	0.08	0.48	0.26	0.34	0.17	0.48	0.464^NS^
41	0.11	0.52	0.25	0.28	0.09	0.37	0.448^NS^
31	0.14	0.45	0.08	0.51	0.10	0.46	0.903^NS^
32	0.03	0.42	0.25	0.44	0.20	0.49	0.049^∗^

NS: not significant, ^∗^ *p* < 0.05.

One-way ANOVA, post hoc Tukey test.

**Table 4:** Correlations among different factors (Pearson correlation coefficients and significance levels).

	Inc (Tb to Ta)	42 (Tb to Ta)	41 (Tb to Ta)	31 (Tb to Ta)	32 (Tb to Ta)	Inclination group
Inc (Tb to Ta)	—					
42 (Tb to Ta)	0.080^NS^	—				
41 (Tb to Ta)	0.019^NS^	0.499^∗∗^	—			
31 (Tb to Ta)	0.025^NS^	0.449^∗∗^	0.672^∗∗^	—		
32 (Tb to Ta)	0.151^NS^	0.576^∗∗^	0.547^∗∗^	0.462^∗∗^	—	
Inclination groups	0.833^∗∗^	0.075^NS^	−0.038^NS^	−0.033^NS^	0.207^∗^	—

NS: not significant, ^∗^ *p* < 0.05, ^∗∗^ *p* < 0.001, and Pearson correlation test.
